# Exclusive and Dual Cigarette and Hookah Smoking Is Associated with Adverse Perinatal Outcomes among Pregnant Women in Cairo, Egypt

**DOI:** 10.3390/ijerph182412974

**Published:** 2021-12-09

**Authors:** Omar El-Shahawy, Kareem Labib, Elizabeth Stevens, Linda G. Kahn, Wagida Anwar, Cheryl Oncken, Tom Loney, Scott E. Sherman, Erin L. Mead-Morse

**Affiliations:** 1 Department of Population Health, New York University School of Medicine, New York, NY 10016, USA; Elizabeth.Stevens@nyulangone.org (E.S.); scott.sherman@nyulangone.org (S.E.S.); 2 School of Global Public Health, New York University, New York, NY 10013, USA; 3 Department of Obstetrics and Gynecology, Faculty of Medicine, Ain Shams University, Cairo 11517, Egypt; kareem_labib@yahoo.com; 4 Departments of Pediatrics and Population Health, New York University School of Medicine, New York, NY 10016, USA; Linda.Kahn@nyulangone.org; 5 Department of Community and Environmental Medicine, Faculty of Medicine, Ain Shams University, Cairo 11517, Egypt; wagidaanwar@gmail.com; 6 Department of Medicine, University of Connecticut Health Center, Farmington, CT 06030, USA; oncken@uchc.edu (C.O.); mead@uchc.edu (E.L.M.-M.); 7 College of Medicine, Mohammed Bin Rashid University of Medicine and Heath Sciences, Dubai P.O. Box 505055, United Arab Emirates; tom.loney@mbru.ac.ae

**Keywords:** birth outcomes, Egypt, fetal growth retardation, hookah, smoking, pregnancy, smoking water pipes

## Abstract

This study assessed the prevalence of prenatal smoking, factors associated with prenatal smoking, and its association with birth outcomes in a sample of pregnant women in Egypt. Pregnant women were recruited during their last trimester from antenatal clinics in Cairo from June 2015 to May 2016. Participants completed an interviewer-administered survey that assessed tobacco use and attitudes, and exhaled carbon monoxide (CO) was measured. Gestational age at delivery and offspring birth weight were collected via a postnatal phone interview. Two hundred pregnant women ages 16–37 years participated. More than a quarter (29.0%) of women reported smoking (cigarettes, hookah, or both) during their current pregnancy, and hookah was more popular than cigarettes. Most women who smoked prior to their current pregnancy either maintained their current smoking habits (46.6%) or switched from dual to hookah-only smoking (46.6%). Current smokers during pregnancy had a higher mean (±SD) exhaled CO level (2.97 ± 1.45 vs. 0.25 ± 0.60 ppm, *p* < 0.001) and had babies with a lower mean birth weight (2583 ± 300 vs. 2991 ± 478 g, *p* < 0.001) than non-smokers. Smokers during pregnancy had greater odds of premature birth and/or low birth weight babies compared to non-smokers. Dual cigarette-hookah smokers had the highest risk. Additional focused programs are required to prevent women of childbearing age from initiating tobacco use and empower women to stop tobacco use during the preconception and gestational periods.

## 1. Introduction

Use of combustible tobacco during pregnancy is one of the most important modifiable causes of poor maternal and fetal health outcomes worldwide [1,2]. Maternal cigarette and hookah smoking are associated with an increased risk of adverse pregnancy and birth outcomes [3,4]. The most consistent effect is an increased risk of preterm birth (PTB, <37 gestational weeks) and low birth weight (LBW, <2500 g) [1,4,5,6,7]. Extremely preterm babies (<28 weeks) or neonates suffering from LBW, particularly those weighing less than 1500 g or deemed very LBW, have much greater morbidity (e.g., cardio-respiratory complications) and mortality than normal weight infants [8,9]. Smoking cessation during pregnancy can moderate these outcomes. By 16 weeks gestation [10,11], or even as late as the third trimester [11], cessation can result in a near-normal birth weight infant. Even reduced smoking can improve birth weight [12]. Nevertheless, evidence from several countries suggests that the majority of women who are smoking when they become pregnant continue to smoke throughout pregnancy [13,14,15].

The global tobacco epidemic continues to shift from high-income countries to low- and middle-income countries, with an increasing burden among women [16]. Egypt, a lower middle-income country [17], is one of the highest consumers of tobacco in the Middle East and North Africa region [18,19]. Cigarettes are the main form of combustible tobacco used in Egypt, followed by hookah (a water pipe instrument used for smoking tobacco that is usually flavored), which is locally referred to as shisha [20]. The prevalence of smoking among adult men is estimated to be 37.7% for cigarettes and 3.3% for hookah according to the latest Global Adult Tobacco Survey (GATS) that was conducted in 2009 [21]. Smoking among women is estimated to be 0.5% for cigarettes and 0.3% for hookah, and smoking among pregnant women is estimated to be 0.4% for overall combustible tobacco [22]. However, the GATS utilizes a household sampling technique that could underreport women’s smoking given privacy concerns and the cultural stigma against women’s smoking [21,23,24]. Therefore, there is a need for more reliable and recent data on smoking among pregnant women in Egypt.

A prior study estimated smoking among a convenience sample of college-age women recruited from hookah cafes around campuses to be up to 40%, which highlights the growing appeal of smoking among young women of reproductive age in Egypt [25]. While this does not reflect the population prevalence of hookah smoking, this finding is consistent with the literature supporting increasing smoking rates among women in settings where smoking could be taken as a sign of independence, such as urban colleges [24,26]. There is also anecdotal evidence from several gynecologists and obstetricians practicing within Egypt (personal communication: coauthors KL and WA) that in their clinics, they often encounter women of childbearing age who smoke.

There are no published studies that assess the prevalence and factors associated with smoking behavior among pregnant women in Egypt. This information is important in order to guide tobacco control efforts in the country and to assess the need for targeted intervention among pregnant women. To fill this gap, the purpose of this study was to estimate the prevalence of cigarette and hookah smoking, how tobacco use patterns change during pregnancy, attitudes and knowledge about tobacco use, and factors associated with smoking among a sample of pregnant women in the Cairo metropolitan area. As a secondary aim, we examined the associations of cigarette and hookah smoking during pregnancy with gestational age at delivery and birth weight.

## 2. Materials and Methods

### 2.1. Setting and Population

Pregnant women were recruited during their last trimester from antenatal clinics in one of the largest public maternity hospitals and two private obstetric clinics in Cairo, Egypt, from June 2015 to May 2016. These settings were selected to ensure diversity in the women’s demographics, including area of residence and education. The public hospital has a wide catchment area and primarily serves pregnant women of low to middle socioeconomic status. Private clinics usually provide care to pregnant women of middle to high socioeconomic status, as the clinics require either private insurance or out-of-pocket payment. There is a lack of data on cigarette and water pipe smoking among pregnant women in Egypt; however, previous work on pregnant women in Lebanon reported 17–23% cigarette smoking and 4–6% water pipe smoking during pregnancy [27,28,29]. Assuming a conservative prevalence estimate of prenatal tobacco use of ~15% among Egyptian women, a sample of 196 would provide an estimated population prevalence of between 10% and 20% with 95% confidence.

Pregnant women were approached in the waiting rooms of the clinics and asked, “Do you want to participate in a survey regarding your opinions of tobacco smoking during pregnancy?” We approached a total of 229 pregnant women in the waiting rooms of the clinics during office hours, following a convenience sampling approach. Those who agreed to participate were screened for eligibility. Women were eligible if they were pregnant in their third trimester and had no reported complications in their current pregnancy. The participants were informed that their responses would remain confidential and they would be contacted within 10–14 days after their expected date of delivery to ask about the birth weight and gestational age of the baby at birth. All surveys were interviewer-administered and completed by two co-authors (KL and WA). The surveys took approximately 10–15 min to complete. Women with chronic medical conditions before or during pregnancy such as hypertension, diabetes mellitus, heart disease, and renal disease, as well as women who were believed to be mentally or physically incapable of participating in the interview, as judged by the interviewer, were excluded from the sample. This study was approved by the Institutional Review Board of Ain Shams University. Appropriate permissions were obtained from participating clinics.

### 2.2. Measures

The questions used in the study were adapted from Bloch et al. [30], who assessed secondhand smoke (SHS) exposure and tobacco use among pregnant women in low- and middle-income countries. This adaption process was led by CO and other authors (OS, EM, KL) with international and local expertise in tobacco use surveys. All participants answered questions about their sociodemographic characteristics, expected date of delivery, and beliefs about tobacco use, as well as about SHS exposure and tobacco use by themselves, their husbands, and other household members. We collected age (years), educational level [preparatory school completed or less (<10 years), some secondary school or greater (≥10 years)], employment status (employed or not employed), and residential area (urban, suburban, rural).

### 2.3. Smoking Status

Participants were asked how often they had used each of the following tobacco products before this current pregnancy: hookah, cigarettes, electronic cigarettes, and other products. Response options included never, yearly, monthly, weekly, or daily. The same question was asked about their use during this current pregnancy at the time of the interview. Participants were considered a smoker of a tobacco product if they reported using it daily, weekly, or monthly, and a non-smoker if they reported using it yearly or never. Based on their smoking status before and during pregnancy, we categorized participants according to changes in their smoking behaviors of any tobacco product(s): non-smokers (women who were non-smokers before and during their current pregnancy), quitters (women who were smokers before pregnancy and non-smokers during their current pregnancy), maintainers (women who smoked before pregnancy and maintained smoking during their current pregnancy), switchers from dual to hookah-only smoking, switchers from dual to cigarette-only smoking, and switchers from a single product (either cigarette or hookah) to dual smoking. There were no switchers from cigarette-only to hookah-only or from hookah-only to cigarette-only in our sample.

### 2.4. Tobacco Use History

Current smokers were asked about their history of tobacco use, motivation to quit tobacco use, self-efficacy to quit tobacco use, and intentions to quit smoking cigarettes and/or hookah. Motivation to quit was assessed with the following item: “How motivated are you to quit all tobacco use during your current pregnancy?” Self-efficacy to quit was assessed with the following item: “How confident are you that you are able to quit all tobacco use during your current pregnancy?” Both items were measured on an 11-point Likert scale (0 = not at all, 10 = extremely). Intention to quit using cigarettes and/or hookah was assessed with the following items: “Do you intend to quit [using a hookah/smoking cigarettes] at any time?” (not at all, in the next month, in the next 6 months, in the future). Hookah smokers were asked how many bowls of tobacco they smoked using a hookah in the past month, and cigarette smokers were asked the number of days they smoked cigarettes in the past month and, on days they smoked, how many cigarettes they smoked on average. Smokers were also asked how long they had used a hookah/smoked cigarettes at this frequency (less than 6 months, 6 months to <1 year, 1 to <2 years, 2 years to <3 years, 3 years to <4 years, 4 years or longer).

### 2.5. Attitudes toward and Knowledge about Tobacco

To assess attitudes toward tobacco use, all participants were asked how much they agreed or disagreed with the following statements: (1) “It is socially acceptable for women to smoke cigarettes”, (2) “It is socially acceptable for women to smoke shisha [hookah]”, (3) “It is easy to tell people living with you not to smoke at home”, and (4) “It is easy to tell guests visiting you not to smoke at home”. To assess knowledge of the harms of tobacco, participants were asked how much they agreed or disagreed with the following statements: (5) “A pregnant woman’s use of tobacco (shisha [hookah], cigarettes, etc.) is harmful to her or her unborn baby’s health”, (6) “A pregnant woman’s exposure to tobacco smoke of someone else is harmful to her or her unborn baby’s health”, and (7) “Tobacco smoke exposure is harmful to a newborn’s health”. All responses were on a 5-point Likert scale (1 = very much disagree, 2 = slightly agree, 3 = neither agree nor disagree, 4 = slightly agree, 5 = very much agree). Questions 1 and 2 were averaged to create perceived social acceptability of smoking (Cronbach’s α = 0.99), and questions 3 and 4 were averaged to create perceived ease of telling others not to smoke at home (Cronbach’s α = 0.72). Items 5–7 were kept as separate items because of poor inter-item reliability.

### 2.6. Biological Outcomes

Exhaled carbon monoxide (CO) was collected at the time of the survey using Covita (piCo, now named Micro-basic) Smokerlyzer. We collected infants’ gestational age (weeks) and birth weight (grams) at delivery from participants during a follow-up phone call 10–14 days post-delivery.

### 2.7. Analysis

We examined sociodemographic characteristics in the total sample and by current smoking status. Differences in smoking characteristics were examined by the type of product currently smoked (hookah-only, cigarette-only, or dual hookah and cigarette smoking). We also explored patterns of cigarette and hookah smoking before and during pregnancy, and assessed factors associated with changes in smoking behaviors. To test for group differences, we used Wilcoxon–Mann–Whitney, Fisher’s exact, and Pearson’s χ^2^ tests.

We used multivariable logistic regression to examine the odds of current smoking during pregnancy as a function of age, education, urban residence (vs. suburban/rural residence), and husband’s smoking status. We did not adjust for employment status because: (1) a post-estimation Wald χ^2^ test showed that removing the variable from the model would not harm model fit, and (2) the variable had 11 missing values. Moreover, we combined suburban and rural residential locations into one reference category because all women in the sample living in rural areas were non-smokers. Due to small sample sizes, we were not able to stratify by type of product smoked (cigarette, hookah, or both). We conducted multivariable regression of the odds of PTB (<37 weeks) and/or LBW (<2500 g) by smoking status and product use, adjusted for age, education, residential location, and husband’s smoking status. PTB and LBW were combined due to the small sample size. All analyses were performed using Stata 16.1. Adjusted odds ratios (aOR) with 95% confidence intervals (CI) were reported. Statistical significance was defined by a *p*-value < 0.05.

## 3. Results

### 3.1. Sociodemographic Characteristics and Pattern of Cigarette and Hookah Smoking

A total of 200 pregnant women ages 16–37 years were enrolled (87% response rate), all of whom provided follow-up information after delivery. About half (53%) were older than 26 years (Table 1). Approximately half (52%) had at least secondary school or higher education (≥10 years of schooling), and the majority (77%) were not employed. Nearly half of women (44%) were living in rural areas, 18% were living in suburban areas, and 38% were living in urban areas. About two-thirds (69%) of women had a husband who smoked tobacco. Of these women, most reported that their husbands predominantly smoked cigarettes (88%), followed by hookah (12%). Before their current pregnancy, most women reported being non-smokers (70.0%), followed by dual cigarette and hookah smoking (19.0%), cigarette-only smoking (9.5%), and hookah-only smoking (1.5%). During their current pregnancy, most women reported being non-smokers (71.0%), followed by hookah-only smoking (14.5%), cigarette-only smoking (10.0%), and dual smoking (4.5%). Only one woman reported e-cigarette use before pregnancy and none reported it during pregnancy. Table 1 presents differences in sociodemographic characteristics and history of smoking behavior by current smoking status (cigarettes and/or hookah). Smokers were older, more highly educated, more likely to live in urban areas, and more likely to have husbands who smoked. Only two women who smoked before pregnancy reported quitting during their current pregnancy. Most of the women who smoked prior to their current pregnancy either maintained their current smoking habits (46.6%) or switched from dual to hookah-only smoking (46.6%). Very few pregnant women switched from dual to cigarette-only smoking (5.2%) or from single product to dual smoking (1.7%), and none switched from cigarette-only to hookah-only or vice versa.

### 3.2. Smoking Characteristics by Product Type

Supplemental Table S1 presents smoking history and characteristics by type of product smoked. Most hookah-only smokers during pregnancy reported smoking hookah daily (93.1%), followed by weekly (6.9%), whereas most dual smokers during pregnancy reported smoking hookah monthly (66.7%), followed by weekly (22.2%) and daily (11.1%, *p* < 0.001). Hookah-only smokers reported smoking more bowls of tobacco in the past month, on average, than dual smokers (9.7 ± 1.2 vs. 4.0 ± 3.7, *p* < 0.001) and smoking hookah longer at their current frequency than dual smokers (93.1% of hookah-only smokers reported smoking for 3–4 years vs. 75.0% of dual smokers reported smoking for less than 6 months, *p* < 0.001).

Smoking characteristics did not significantly differ between cigarette-only and dual smokers during pregnancy. Most cigarette-only smokers during pregnancy reported smoking cigarettes daily (55.0%), followed by monthly (30.0%) and weekly (15.0%). Similarly, most dual smokers during pregnancy reported smoking cigarettes daily (77.8%), followed by weekly (22.2%). On average, cigarette-only smokers reported smoking cigarettes on 16.5 (±13.2) days in the past month and 4.9 (±4.4) cigarettes per day on days smoked, and dual smokers reported smoking cigarettes on 24.4 (±11.0) days in the past month and 3.3 (±2.6) cigarettes per day on days smoked. More than half of cigarette-only smokers (55.0%) and two-thirds of dual smokers (66.7%) reported smoking cigarettes at their current frequency for less than 6 months.

Intentions to quit smoking were low in all groups. Most hookah-only (96.6%) and all dual smokers stated that they intended to quit hookah smoking “sometime in the future,” with no statistically significant difference. Dual smokers had slightly higher intentions to quit cigarette smoking than cigarette-only smokers. More than half of dual smokers reported intentions to quit cigarettes within the next 6 months (55.6%) followed by “sometime in the future” (44.4%), compared with nearly all cigarette-only smokers reporting “sometime in the future” (95.0%, *p* = 0.001). Only one hookah-only and one cigarette-only smoker reported no intention to quit.

### 3.3. Attitudes, Knowledge, and Motivation and Self-Efficacy to Quit

To better understand women who switched from dual use to exclusive hookah smoking, we examined knowledge and attitudes about smoking, and motivation and self-efficacy to quit among women with the three most common behaviors: non-smokers (i.e., women who were non-smokers before and during their current pregnancy), maintaining smokers (i.e., women who did not change their smoking habits from before to during their current pregnancy), and switchers (i.e., dual smokers who switched to hookah-only smoking during pregnancy). Two women who quit smoking, three women who switched from dual to cigarette-only, and one woman who switched from cigarette-only to dual smoking were excluded. Results are presented in Table 2.

Compared with non-smokers, maintaining smokers and switchers perceived women’s smoking to be more socially acceptable and were less knowledgeable that smoking during pregnancy harms the mother and fetus. Switchers thought it was easier to enforce non-smoking rules at home than both non-smokers and maintaining smokers. Non-smokers had the highest level of knowledge about the harm of SHS exposure to newborn health, followed by maintaining smokers. Switchers had higher motivation and self-efficacy to quit than maintaining smokers.

### 3.4. Sociodemographic Factors Associated with Smoking during Pregnancy

Table 3 presents the results of the multivariable regression of the odds of current smoking during pregnancy (cigarette-only, hookah-only, or dual smoking) as a function of age, education, residential area, and husband’s smoking status. Women with greater education (aOR: 26.76, 95% CI: 5.48, 130.62) and living in urban areas (aOR: 19.98, 95% CI: 6.31, 63.25) had greater odds of smoking than those with less education and living in suburban or rural areas. Age and husband’s smoking status were not associated with women’s smoking status.

### 3.5. Carbon Monoxide and Perinatal Outcomes by Tobacco Exposure

Table 4 shows the unadjusted difference in mean CO, gestational age, and birth weight by type of product smoked. Hookah-only smokers had a higher mean CO than cigarette-only smokers (3.17 ± 1.07 vs. 2.65 ± 1.90 ppm, *p* = 0.032), but there was no difference between exclusive product and dual smoking (3.00 ± 1.41 ppm). Cigarette-only smokers had babies with lower mean birth weight than dual smokers (2645 ± 196 vs. 2478 ± 164 g, *p* = 0.048), but there was no difference between cigarette-only and hookah-only smokers (2572 ± 181 g) or between hookah-only and dual smokers. Hookah-only smokers delivered later (37.6 ± 1.1 weeks) than cigarette-only (36.8 ± 0.6 weeks, *p* = 0.005) and dual (36.7 ± 0.8 weeks, *p* = 0.024) smokers, but gestational age at birth did not differ between cigarette-only and dual smokers. Non-smokers had lower mean CO levels (0.25 ± 0.60 ppm) and higher birth weight (2990.49 ± 477.47 g) and gestational age (38.49 ± 1.43 weeks) than hookah-only smokers, cigarette-only smokers, and dual smokers (*p* < 0.001).

In the adjusted multivariable regression (Table 5), the odds of LBW and/or PTB were higher among smokers (aOR: 13.49, 95% CI: 3.54, 51.33) and women whose husbands smoked (aOR: 3.87, 95% CI: 1.36, 10.99), and lower among women with higher education (aOR: 0.25, 95% CI: 0.08, 0.81). Looking at type of products smoked, hookah-only (aOR: 9.55, 95% CI: 2.17, 42.00), cigarette-only (aOR: 13.57, 95% CI: 3.14, 58.60), and dual (aOR: 40.06, 95% CI: 5.30, 302.59) smokers had greater odds of LBW and/or PTB than non-smokers, adjusting for age, education, urban residence, and husband’s smoking status (Table 6).

## 4. Discussion

This study represents the findings from a cross-sectional survey with a follow-up to birth among a convenience sample of pregnant women attending antenatal care clinics in Cairo, Egypt to analyze the association of smoking cigarettes and hookah with pregnancy and birth outcomes. More than a quarter of women reported smoking (cigarettes, hookah, or both) during their current pregnancy. Most women who smoked prior to their current pregnancy either maintained their current smoking habits or switched from dual to hookah-only smoking. Current smokers during pregnancy (cigarettes, hookah, or both) had a higher mean exhaled CO, were more likely to experience PTB, and had babies with a lower mean birth weight than non-smokers. These findings are particularly novel in accounting for birth outcomes among pregnant women who smoke hookah, a population with a paucity of data in the literature.

These findings suggest that the prevalence of tobacco use among pregnant women in Cairo, Egypt might be higher than the current published estimates as represented through the most recent GATS and warrant further evaluation, particularly among urban and educated women. The finding that nearly 30% of pregnant women smoked, as compared to the GATS 2010 estimate of <1% [22], raises potential concerns that surveys using a household sampling approach might significantly underestimate the number of women who smoke during pregnancy, potentially because this behavior is not socially acceptable in that setting. Indeed, within the Egyptian culture, where traditional gender roles depict women’s smoking as disrespectful to society, women often keep their smoking secret from their families [24]. Household surveys pertaining to sensitive behaviors, such as smoking among women in Egyptian culture, are subject to significant underreporting [21,23,24]. A study by El-Shahawy et al. (2018) [31] suggests that the smoking rates among adolescent girls in Egypt are almost ten times higher than reported estimates from the GATS [22]. There is no reason to expect these rates would dramatically drop when women reach childbearing age, as generally tobacco smoking cessation rates in most countries remain low [30] and adolescent smoking habits generally track into adulthood [32,33,34]. Another interpretation is that the rates in a major city, such as Cairo, may be substantially higher than the rest of Egypt, resulting in the low national overall prevalence indicated in GATS. Furthermore, this study revealed that few women quit smoking when pregnant, as most of the women who smoked prior to their current pregnancy either maintained their current smoking habits or switched from dual to hookah-only smoking. In Egypt, improved tobacco control policies and public health interventions are needed to decrease tobacco use and encourage cessation during pregnancy to improve maternal and fetal outcomes. While Egypt has ratified the Framework Convention on Tobacco Control, the antismoking legislation that was last updated in 2002 (and includes smoking bans in public places and restaurants) is not fully enforced [20,35,36]. Authors were not able to find any published data on major campaigns or interventions targeting exposure to SHS among pregnant women. Additional outreach efforts are needed to reduce the rate of smoking during pregnancy among Egyptian women, with particular emphasis on educated women in urban areas.

Targeted interventions are needed to educate women of childbearing age, and the population in general, about the health risks of hookah smoking. Among women who were dual smokers prior to pregnancy, more than 70% switched to smoking hookah alone. Compared with women who continued to smoke but did not switch to hookah, the switchers generally had a higher motivation to quit smoking as well as a higher perceived self-efficacy to quit. Given that switchers and women who maintained their smoking habits did not differ in their knowledge of the harms of smoking, the existing perception that hookah is easier to quit or is used as a way to quit cigarettes could explain why some women switched to hookah [37]. An alternative explanation is that women may have switched as a perceived harm reduction approach. This is consistent with previous research that found hookah smoking is often thought of as safer than cigarettes [37,38,39,40,41]. One limitation of this study is that we cannot ascertain the validity of this hypothesis in this particular sample, as we did not ask about the differences in perceptions between cigarettes and hookah, rather about tobacco use in general. Additionally, women who smoked hookah were less likely than cigarette smokers to change their smoking habits when they became pregnant. A recent prospective cohort study in Iran found that quitting hookah during pregnancy had positive effects on infant growth, especially birth weight [42]. Targeting hookah smokers to encourage smoking cessation or reduction may have a vital impact on birth outcomes [10,11,12].

High levels of tobacco exposure among Egyptian women during pregnancy may represent a barrier to achieving birth and survival outcomes on par with other middle-income countries, which have an average neonatal mortality rate of 7 deaths per 1000 live births, as compared with 11 per 1000 in Egypt [43]. Our study affirmed previous observations that maternal cigarette or hookah smoking is associated with increased risk of LBW [1,4,5,6,7]. Among women in our study, those who smoked during their pregnancy, and dual smokers in particular, experienced significantly greater odds of PTB and/or LBW. Neonates suffering from PTB and LBW have greater morbidity and mortality than normal weight infants [8]. Therefore, to achieve its maternal and infant health targets, Egypt would do well to invest in significant efforts to reduce maternal smoking [44].

This study had a few limitations. Although the sample size was sufficient to achieve study objectives, we had limited power to examine smoking patterns among subpopulations, especially dual users. We also excluded women with any reported complications in their current pregnancy, which might have masked additional adverse outcomes of smoking in pregnancy. The study was primarily cross-sectional and retrospective in nature and relied on self-reported recall of habits before and during pregnancy, capturing participants late in their pregnancy. Therefore, our ability to assess the relations of potentially time-varying measures, such as motivation and self-efficacy to quit, with changes in tobacco use at different stages of pregnancy was limited. Finally, we recruited pregnant women from the largest public maternity hospital and two private obstetric clinics in Cairo to minimize selection bias and maximize the generalizability of the findings. However, using a convenience sampling approach might limit the generalizability of the findings. Additional research examining these associations longitudinally and beginning at earlier stages of pregnancy—or even preconception—may reveal insights that could guide the targeting of anti-tobacco messages to women of reproductive age.

## 5. Conclusions

Maternal smoking, particularly hookah smoking, may be much more prevalent in Egypt than previously estimated. Many women do not intend to quit nor actually quit smoking when they become pregnant. We found that smokers often switched to hookah smoking during pregnancy. Moreover, prenatal smoking was associated with PTB and LBW, especially dual hookah-cigarette smoking. To achieve maternal and infant sustainable development goals, more emphasis on reducing maternal tobacco exposure is needed in Egypt, particularly with regard to hookah smoking. Additional studies are needed to understand pregnant women’s perceptions of harm and addictiveness by tobacco product, thus allowing for better targeted interventions.

## Figures and Tables

**Table 1:** Background characteristics and tobacco use and exposure by current smoking status (cigarettes and/or hookah) in pregnant women in Cairo, Egypt (*N* = 200).

	Non-Smoker During Pregnancy	Smoker During Pregnancy	*p*-Value
	(*n* = 142)	(*n* = 58)	
Background Characteristics			
Age in years,			
mean (SD)	25.97 (4.66)	29.74 (3.45)	
median	25.0	30.0	
range	16–36	22–37	<0.0001 ^1^
Age group, %(*n*)			
16–26 years	58.5% (83)	17.2% (10)	
>26 years	41.5% (59)	82.8% (48)	<0.0001 ^2^
Educational level, %(*n*)			
Preparatory school or less (<10 years)	66.2% (94)	3.5% (2)	
Secondary school or higher (≥10 years)	33.8% (48)	96.5% (56)	<0.0001 ^3^
Residential area, %(*n*)			
Urban	16.2% (23)	91.4% (53)	
Suburban	21.8% (31)	8.6%(5)	
Rural	62.0% (88)	0% (0)	<0.0001 ^3^
Employment status, %(*n*) (*N* = 189)			
Employed	22.1% (29)	24.1% (14)	
Unemployed	77.9% (102)	75.9% (44)	0.762 ^2^
Tobacco Use and Exposure			
Exhaled carbon monoxide (ppm), mean (SD)	0.25 (0.60)	2.97 (1.45)	< 0.0001 ^1^
Smoked before pregnancy, %(*n*)			
None	98.6% (140)	0% (0)	
Hookah only	0.7% (1)	3.5% (2)	
Cigarettes only	0.7% (1)	31.0% (18)	
Both cigarettes and hookah	0% (0)	65.5% (38)	<0.0001 ^3^
Smoked during pregnancy, %(*n*)			
None	100% (142)	--	
Hookah only	--	50.0% (29)	
Cigarettes only	--	34.5% (20)	
Both cigarettes and hookah	--	15.5% (9)	--
Change in smoking behavior from before to during pregnancy, %(*n*)			
Maintained not smoking	98.6% (140)	--	
Quit smoking	1.4% (2)	--	
Maintained smoking pattern	--	46.5% (27)	
Switched from dual product use to hookah-only smoking	--	46.5% (27)	
Switched from dual product use to cigarette-only smoking	--	5.2% (3)	
Switched from single product use to dual product use	--	1.7% (1)	--
Husband smokes, %(*n*) (*N* = 196)			
No	42.5% (59)	3.5% (2)	
Yes	57.5% (80)	96.5% (55)	<0.0001 ^3^
Birth Outcomes			
Gestational age at delivery in weeks, mean (SD)	38.49 (1.43)	37.17 (0.97)	<0.0001 ^1^
Premature birth (<37 weeks), %(*n*)	12.7% (18)	41.4% (24)	<0.0001 ^2^
Birth weight in grams, mean (SD)	2990.49 (477.47)	2582.76 (189.31)	<0.0001 ^1^
Low birth weight (<2500 g), %(*n*)	7.8% (11)	37.9% (22)	<0.0001 ^2^

^1^ Tested using Wilcoxon–Mann–Whitney test. ^2^ Tested using χ^2^ test. ^3^ Tested using Fisher’s exact test.

**Table 2:** Attitudes, knowledge, and quitting motivation and self-efficacy associated with changes in smoking among pregnant women in Egypt (*N* = 194), mean (SD).

	Non-Smokers ^3^ (*n* = 140)	Maintaining Smokers ^4^ (*n* = 27)	Switchers from Dual to Hookah ^5^ (*n* = 27)
It is socially acceptable for women to smoke ^1^	1.23 (0.61) ^a,b^	4.87 (0.43) ^a^	4.83 (0.24) ^b^
It is easy to tell others not to smoke at home ^1^	2.24 (1.06) ^a^	2.43 (1.30) ^b^	3.80 (0.35) ^a,b^
A pregnant woman’s use of tobacco is harmful to her or her unborn baby’s health ^1^	4.48 (0.90) ^a,b^	4.07 (0.87) ^a^	4.15 (0.36) ^b^
A pregnant woman’s exposure to tobacco smoke of someone else is harmful to her or her unborn baby’s health ^1^	3.64 (0.99)	3.85 (0.53)	4.00 (0.00)
Tobacco smoke exposure is harmful to a newborn’s health ^1^	4.57 (0.86) ^a,b^	4.26 (0.59) ^a,c^	4.00 (0.00) ^b,c^
Motivation to quit all tobacco use during current pregnancy ^2^	--	3.44 (1.29) ^a^	5.00 (0.00) ^a^
Self-efficacy to quit all tobacco use during current pregnancy ^2^	--	3.92 (1.00) ^a^	5.00 (0.00) ^a^

^1^ Attitudes and knowledge were measured on a 5-point Likert scale: 1 = very much disagree, 5 = very much agree. ^2^ Motivation and self-efficacy to quit were only asked of current smokers, and were measured on an 11-point Likert scale: 0 = not at all, 10 = extremely. ^3^ Non-smokers: women who were non-smokers before pregnancy and remained non-smokers during their current pregnancy. ^4^ Maintaining smokers: women who kept the same smoking habits before and during pregnancy (i.e., cigarette-only smokers remained cigarette-only smokers, hookah-only smokers remained hookah-only smokers, and dual smokers remained dual smokers). ^5^ Switchers: women who switched from dual smoking before pregnancy to hookah-only smoking during their pregnancy. ^a,b,c^ Within each row, means with the same superscript letter are significantly different from each other at *p* < 0.05.

**Table 3:** Multivariable analysis of sociodemographic factors associated with current smoking (cigarettes and/or hookah) during pregnancy in Cairo, Egypt (*N* = 196).

	Odds Ratio	95% CI	*p*-Value
Age group			
16–26 years	Ref		
>26 years	2.03	0.61, 6.79	0.252
Educational level			
Preparatory school or less (<10 years)	Ref		
Secondary school or higher (≥10 years)	26.76	5.48, 130.62	<0.0001
Residential area			
Suburban/Rural	Ref		
Urban	19.98	6.31, 63.25	<0.0001
Husband smokes			
No	Ref		
Yes	4.52	0.74, 27.75	0.104

**Table 4:** Carbon monoxide and prenatal outcomes by product use during pregnancy in Egypt (*N* = 200), mean (SD).

	Non-Smokers (*n* = 142)	Hookah-Only Smokers (*n* = 29)	Cigarette-Only Smokers (*n* = 20)	Dual Smokers (*n* = 9)
Carbon monoxide (CO), mean ± SD (ppm)	0.25 ± 0.60 ^a^	3.17 ± 1.07 ^b^	2.65 ± 1.90 ^b^	3.00 ± 1.41
Gestational age at birth, mean ± SD (weeks)	38.49 ± 1.43 ^a^	37.59 ± 1.07 ^c^	36.81 ± 0.61 ^c^	36.65 ± 0.77 ^c^
Birth weight, mean ± SD (grams)	2990.49 ± 477.47 ^a^	2572.41 ± 181.06	2645.00 ± 195.95 ^d^	2477.78 ± 164.15 ^d^

^a^ Non-smokers had statistically significantly lower mean CO, and higher mean birth weight and gestational age than hookah-only smokers, cigarette-only smokers, and dual smokers (*p* < 0.001). ^b^ Hookah-only smokers had a statistically significantly higher mean CO than cigarette-only smokers (*p* < 0.05). ^c^ Hookah-only smokers had a statistically significantly higher mean gestational age at birth than cigarette-only smokers (*p* < 0.01) and dual smokers (*p* < 0.05). ^d^ Cigarette-only smokers had a statistically significantly higher mean birth weight than dual smokers at *p* < 0.05.

**Table 5:** Multivariable analysis of smoking during pregnancy and sociodemographic factors associated with low birth weight and/or preterm birth outcomes in Cairo, Egypt (*N* = 196).

	Odds Ratio	95% CI	*p*-Value
Smoking status during pregnancy			
Non-smoker	Ref		
Smoker	13.49	3.54, 51.33	<0.0001
Age group			
16–26 years	Ref		
>26 years	0.59	0.26, 1.34	0.209
Educational level			
Preparatory school or less (<10 years)	Ref		
Secondary school or higher (≥10 years)	0.25	0.08, 0.81	0.020
Residential area			
Suburban/Rural	Ref		
Urban	1.05	0.36, 3.09	0.929
Husband smokes			
No	Ref		
Yes	3.87	1.36, 10.99	0.011

**Table 6:** Multivariable analysis of product use during pregnancy and sociodemographic factors associated with low birth weight and/or preterm birth outcomes in Cairo, Egypt (*N* = 196).

	Odds Ratio	95% CI	*p*-Value
Product use during pregnancy			
Non-smoker	Ref		
Hookah-only smoker	9.55	2.17, 42.00	0.003
Cigarette-only smoker	13.57	3.14, 58.60	<0.001
Dual user	40.06	5.30, 302.59	<0.001
Age group			
16–26 years	Ref		
>26 years	0.55	0.24, 1.26	0.158
Educational level			
Preparatory school or less (<10 years)	Ref		
Secondary school or higher (≥10 years)	0.25	0.08, 0.81	0.021
Residential area			
Suburban/Rural	Ref		
Urban	1.13	0.38, 3.35	0.824
Husband smokes			
No	Ref		
Yes	3.89	1.37, 11.11	0.011

## Data Availability

The de-identified data presented in this study are available on request from the corresponding author.

## Supplementary Materials

The following are available online at https://www.mdpi.com/article/10.3390/ijerph182412974/s1, Supplemental Table S1. Smoking characteristics by product type in pregnant women smokers (*N* = 58).
