# Supplementary material for: Exclusive and Dual Cigarette and Hookah Smoking Is Associated with Adverse Perinatal Outcomes among Pregnant Women in Cairo, Egypt
Source: Int J Environ Res Public Health. 2021 Dec 9;18(24):12974. doi: 10.3390/ijerph182412974 (PMC8701206; doi:10.3390/ijerph182412974)
Supplement: Supplementary file 1 [file ijerph-18-12974-s001.zip › ijerph-1388318-supplementary.pdf]

International Journal of
Environmental Research
and Public Health

MDPI

Supplemental Table S1. Smoking characteristics by product type in pregnant women smokers (*N* = 58).

	Cigarette-Only Smokers (*n* = 20)	Hookah-Only Smokers (*n* = 29)	Dual Smokers (*n* = 9)
Frequency of cigarette smoking, %(n)			
Monthly	30.0% (6)	--	0% (0)
Weekly	15.0% (3)	--	22.2% (2)
Daily	55.0% (11)	--	77.8% (7)
Frequency of hookah smoking, %(n)			
Monthly	--	0% (0)	66.7% (6)
Weekly	--	6.9% (2)	22.2% (2)
Daily	--	93.1% (27)[a]	11.1% (1)[a]
# of days of cigarette smoked in past month, mean (SD)	16.5 (13.2)	--	24.4 (11.0)
# of cigarettes smoked per day, on days smoked, mean (SD)	4.9 (4.4)	--	3.1 (2.6)
# of bowls of tobacco smoked using hookah in past month, mean (SD)	--	9.7 (1.2)[a]	4.0 (3.7)[a]
How long smoking cigarettes at current frequency, %(n)			
<6 months	55.0% (11)	--	66.7% (6)
6 months to <1 year	0% (0)	--	11.1% (1)
1 to <2 years	0% (0)	--	11.1% (1)
2 to <3 years	30.0% (6)	--	0% (0)
3 to <4 years	15.0% (3)	--	11.1% (1)
4 years or longer	0% (0)	--	0% (0)
How long using hookah at current frequency, %(n)			
<6 months	--	6.9% (2)	75.0% (6)
6 months to <1 year	--	0% (0)	12.5% (1)
1 to <2 years	--	0% (0)	0% (0)
2 to <3 years	--	0% (0)	0% (0)
3 to <4 years	--	93.1% (27)	12.5% (1)
4 years or longer	--	0% (0)[a]	0% (0)[a]
Intentions to quit smoking cigarettes, %(n)			
Not at all	5.0% (1)	--	0% (0)
In the next month	0% (0)	--	0% (0)
In the next 6 months	0% (0)	--	55.6% (5)
In the future	95.0% (19)[a]	--	44.4% (4)[a]
Intentions to quit smoking hookah, %(n)			

Not at all	--	3.5% (1)	0% (0)
In the next month	--	0% (0)	0% (0)
In the next 6 months	--	0% (0)	0% (0)
In the future	--	96.5% (28)	100% (8)

[a] Within each row, values with the same superscript letter are significantly different from each other at $p<0.05$.
